# Can Timi Risk Score Predict Angiographic Involvement in Patients with St-Elevation Myocardial Infarction?

**Published:** 2010

**Authors:** Allahyar Golabchi, Masoumeh Sadeghi, Hamid Sanei, Mohammad Reza Akhbari, Seied Mostafa Seiedhosseini, Pejman Khosravi, Ali Reza Alisaeedi

**Affiliations:** 1 Cardiology Resident, Isfahan University of Medical Sciences, Isfahan, Iran; 2 Associated Professor of Cardiology, Isfahan Cardiovascular Research Center, Isfahan University of Medical Sciences, Isfahan, Iran; 3 Associated Professor of Cardiology, Isfahan University of Medical Sciences, Isfahan, Iran; 4 Cardiologist, Isfahan University of Medical Sciences, Isfahan, Iran; 5 Internal resident, Isfahan University of Medical Sciences, Isfahan, Iran

**Keywords:** TIMI Risk Score, Modified Gensini Risk Score, LVEF, STEMI

## Abstract

**BACKGROUND:**

In most studies, the agreeable risk scores for ST-elevation myocardial infarction (STEMI) consist of thrombolytic in myocardial infarction (TIMI) risk score and modified Gensini risk score. Researchers showed significant relations between TIMI with angiography scores in patients with UA/NSTEMI. We studied this relation in patients with STEMI.

**METHODS:**

We studied CCU patients with STEMI hospitalized in several hospitals of Isfahan, Iran from September 2007 to June 2008. Sampling method of 240 patients was random and simple. Exclusion criteria were incomplete history, nonspecific electrocardiogram changes, left bundle branch block and not accomplished angiography or accomplished angiography after 2 months of STEMI. Questionnaire indices collected on the basis of TIMI (0–14 points). Echocardiography and angiography were done and then, we used Gensini (0–400 points) to review films of angiography. Spearman‘s rank test and Pearson correlation coefficient were used to study the relation between these scores.

**RESULTS:**

One hundred and sixty one patients were male and their average age was 60.02 years. Averages of TIMI and Gensini scores were 6.30±2.5 and 120.77±50.4, respectively. Study showed significant relation between TIMI, age and LVEF (P<0.001, r=−0.46). Also, between Gensini and age, gender and LVEF significant relation was found (P<0.001). But, a meaningful correlation didn't exist between TIMI and the gender (P=0.08). Our study proved direct relation between TIMI risk scores and modified Gensini scores (P<0.001, r=0.55).

**CONCLUSION:**

We may decide quickly and correctly in emergency room to distinguish which patients with STEMI could derive a benefit from invasive strategies using TIMI score. Also, TIMI risk score can be a good predictor to determine the extension of coronary artery disease in patients with STEMI. As a result, we suggest determination of TIMI score for any patient entered emergency room. Also, this score should be recorded at the time patient's discharge.

## Introduction

Almost one million of Americans' population suffer from acute myocardial infarction yearly and 1/3 of ST-elevation myocardial infarction (STEMI) lead to death.^1^ Despite enormous development in diagnosis and management of STEMI in the past four decades, it is still an important health problem in developing countries.^2^ Acute myocardial infarction (MI) is a life-threatening situation and rapid and correct decision making for life saving of patients in emergency room is very important.^3^ Primary treatment for patients with acute STEMI is fibrinolysis or primary angioplasty.^1^ Multiple studies showed that primary angioplasty is better than fibriniolysis, but all patients don't derive benefit from invasive strategies, so we need risk scores to help us classify the patients.^4^ Multiple diagnostic-therapeutic algorithms and scoring systems publicized for patients with STEMI. Their application depends on signs of disease, therapeutic contraindications and hemodynamic situation.^5^ An excellent scoring system should have a high power of prediction, being available and can simply extract correct information in short time in clinical situation. These scores are based on point scores and sum of the scores correlates with the level of risk of disease. Using this method can guide us to decide rapidly for patient's triage in emergency room.^6^ Harrell et al study showed that risk scores should be used to collect information for correct diagnostic-therapeutic planning.^7^ TIMI (thrombolytic in myocardial infarction) risk score has been shown to be useful in different studies. TIMI score has relation with cardiovascular risk events and it is better than electrocardiogram changes or troponin test alone.^8^ Angiography is a gold standard diagnostic method to determine coronary artery stenosis due to atherosclerosis and can show the best anatomical information for therapeutic planning.^1^ One method that determines the severity and extension of coronary artery disease (CAD) is modified Gensini score system.^9^ Guo et al explained that Gensini cumulative index was a good scoring system for detection of severity of CAD.^10^ In this study, our purpose was comparing TIMI risk score with modified Gensini risk score in patients with STEMI.

## Materials and Methods

Our research was a cross-sectional study and sampling method was random and simple. Sample volume was calculated as 240 patients. Patients with STEMI were selected from CCU patients of Chamran, Noor and Al-Zahra hospitals from September 2007 to June 2008. Written consent from was taken from all patients. Revised definition of myocardial infarction is typical rise and/or fall of biochemical markers of myocardial necrosis with at least one of the following criteria: ischemic symptoms, development of pathological Q waves in the ECG, ECG changes indicative of ischemia, and imaging evidence of new loss of viable myocardium or new regional wall motion abnormality. Definition of STEMI on electrocardiogram was 1 mv ST segment elevation in limb leads or 2 mv ST segment elevation in precordial leads, at least 2 leads from one level if other ST segment elevation differential diagnoses were ruled out.^11^ Some patients excluded from the study because they didn't have correct history of onset of chest pain, had relative bed rest, had nonspecific electrocardiogram changes or left bundle branch block or NSTEMI suspicious, didn't accomplish angiography for any reason, low quality of angiography films, or accomplished angiography after 2 months of STEMI diagnosis. Patient's information were recorded in a standard questionnaire which included name, age, address, telephone number, job, past history of diabetes mellitus (DM), hypertension (HTN) and angina, time of beginning of pain, blood pressure, pulse rate, cardiorespiratory examination (fine crackle), S_3_ gallop and jugular vein pulse pressure. Electrocardiograms of patients with ST segment elevation were reviewed by an expert cardiologist. TIMI risk score is based on 8 clinical indices rapidly calculated besides the patient's bed. According to TIMI score (0 to 14 points), we can divide patients with ACS to low risk (scores 0 to 4) and high risk (scores>4) ones^12^ (Table 1). Questionnaire's indices were collected on the basis of TIMI risk score according to NRMI_3_ (National Registry of Myocardial Infarction 3) study that confirmed the value of indices in STEMI. After completion of questionnaires, total scores were calculated for all patients.^12^ Coronary angiography was done for all patients because of diagnostic study, during hospitalization or in 2 months after discharge. The technique of Judkins was applied because of rapid, simple, high diagnostic values and low complications.^13^ Any patient with no angiography or angiography after 2 months was excluded from our study. Then, angiography films were checked by 3 cardiologists. Left ventricle ejection fraction (LVEF) was recorded on the basis of ventriculography or echocardiography by one cardiologist before patient's discharge.

**Table 1:** TIMI risk score.

TIMI Indices	Point Score
Age≥75 years	3
74 y≥Age≥65 years	2
History Of DM or HTN or Angina	1
Systolic blood pressure<100 mm Hg	3
Heart rate>100 beat/min.	2
Killip class≥II	2
Body weight>67 Kg	1
Anterior STEMI	1
Time of beginning of pain to treatment>4 hours	1
Total score	0 to 14

We used modified Gensini risk score for review of angiography films. The points were from 0 to 400. In this score, angiographic CAD extension points calculated from stenosis score×segment score.^9^ (Table 2)

**Table 2:** Modified Gensini risk score.

Segment	Score
LM	5
LAD	20
LCX	20
RCA	20
D_1_	10
OM_1_	10
PDA	10
S_1_	5


**Stenosis Percentage**	**Score**

1–49	1
50–74	2
75–99	3
100	4

Data analyzed by SPSS_13_ software Spearman rank correlation was used to study the relation between TIMI risk score and Gensini risk score. Also, Pearson correlation coefficient was used for confirmation.

## RESULTS

In this research from 240 patients with STEMI, 161 (67%) patients were male. The youngest patient was 17 year-old and the oldest one was 83 year-old. Their average age was 60±11.95 years with standard deviation (SD) of 11.95 years. The total number of 126 (52%) patients were treated by thrombolytic agent (streptokinase) or emergent angioplasty. LVEF of patients was 6 to 70 percent with average of 44.5 percent and SD of 12.59. TIMI risk score of patients was 0 to 13 with average of 6.30 and SD of 2.5 (Table 3).

**Table 3:** TIMI risk score distribution.

TIMI Score	0	1	2	3	4	5	6	7	8	9	10	11	12	13
**Number of Patients**	1	3	11	14	29	41	31	39	23	22	13	6	5	2

Gensini risk score of patients was from 0 to 230 with average of 120.77 and SD of 50.4 (Table 4).

**Table 4:** Gensini risk score distribution of patients (Angiogrphic score).

Gensini	0	21	41	61	81	101	121	141	161	181	201	231
Score	20	40	60	80	100	120	140	160	180	200	230	400
**Number of patients**	10	7	21	21	33	31	32	37	23	9	16	0

Our study showed a meaningful relation between TIMI risk score and age of patients (P<0.001) and a meaningful relation between TIMI score group (low risk vs. high risk) with age of patients (P<0.001). But, a meaningful correlation didn't exist between TIMI risk score and gender of patients (P=0.08). A significant negative relation existed between TIMI risk scores and LVEF of patients (P<0.001, r=−0.46). Also, there was a meaningful relation between TIMI risk score groups and LVEF of patients (P<0.00). According to the Spearman coefficient of correlation, there was a meaningful relation between Gensini risk score and age, gender and LVEF of patients (P<0.001) and Pearson correlation confirmed this relation (P<0.001, Table 5).

**Table 5:** Relationship between TIMI Risk Score with Gensini Risk Score, Age and LVEF.

	TIMI Groups	Number	Med	P value
Gensini	Low Risk	58	70 (60.22–79.78)	
Risk	High Risk	182	136.95 (130.68–143.22)	<0.001
Score	Total	240	120.77 (114.35–127.19)	
	Low Risk	58	52.38 (49.32–55.44)	
Age	High Risk	182	62.45 (60.84–64.06)	<0.001
	Total	240	60.02 (58.50–61.54)	
	Low Risk	58	51.50 (48.85–54.15)	
LVEF	High Risk	182	41.68 (39.87–43.50)	<0.001
	Total	240	44.05 (42.45–45.66)	

Our study showed a meaningful relation between TIMI risk score and age of patients (P<0.001) and a meaningful relation between TIMI score group (low risk vs. high risk) with age of patients (P<0.001). But, a meaningful correlation didn't exist between TIMI risk score and gender of patients (P=0.08). A significant negative relation existed between TIMI risk scores and LVEF of patients (P<0.001, r=−0.46). Also, there was a meaningful relation between TIMI risk score groups and LVEF of patients (P<0.00). According to the Spearman coefficient of correlation, there was a meaningful relation between Gensini risk score and age, gender and LVEF of patients (P<0.001) and Pearson correlation confirmed this relation (P<0.001, Table 5).

**Figure 1:**
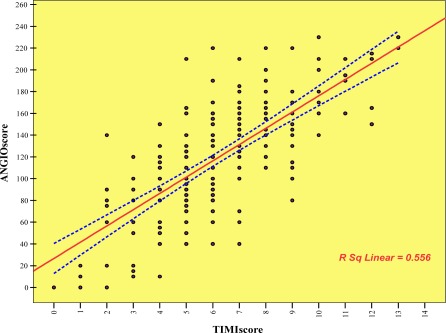
Correlation of prevalence of TIMI risk scores on the basis of modified Gensini risk scores.

Our study showed that the relation of TIMI risk score and modified Gensini risk score was significant on the basis of Spearman correlation (P<0.001) and these findings (Positive relation) were confirmed with Pearson correlation (P <0.001, r=0.55, Figure 1. Also, significant relation existed between TIMI risk score groups and modified Gensini risk score (P<0.001).

## DISCUSSION

We studied correlation between TIMI risk scores with modified Gensini risk scores on the basis of angiography results in patients with STEMI. Also, we evaluated relationship of these two risk scores with age, gender and LVEF of patients. The significant correlation between TIMI risk scores and age of patients was confirmed other researches.^14^ Mandeep et al study showed a real relation between TIMI risk scores and angiography scores with LVEF and age of patients; 8 these results were similar to those of our study. Antman et al study showed that in patients with STEMI, starting the reperfusion therapy without wasting the time is really important and using the scoring system for these patients can be useful. So, we suggest the necessity of application of valid risk scores in management of patients with ACS.

The relationship between TIMI risk score and efficacy of conservative or interventional strategy in patients with non ST-segment elevation ACS in Zhao et al study showed that early invasive strategy may significantly reduce combined cardiovascular events in NSTEMI patients with moderate and high TIMI risk score compared with early conservative strategy.^15^ Walsh et al cleared that PCI can be performed in an elderly, high-risk TIMI score population with a low mortality and marked symptomatic benefit.^16^ In Mathew et al^17^ and Garcia et al^18^ studies, "correlation between clinical risks with extension of CAD in patients suffered from NSTEMI" showed that the most low clinical risk patients had normal angiography or limited CAD, but severe CAD or left main artery disease in high clinical risk patients was more prevalent than that in low risk patients; so the clear relations were existed between TIMI risk score and angiography score in patients with NSTEMI. Studies showed this relation in patients with UA/NSTEMI only but our study confirmed this relation in patients with STEMI; thus the meaningful relation existed between TIMI risk score with modified Gensini risk score. So, TIMI risk score can be an good predictor to determine the extension of CAD and management of patients with ACS in our emergency rooms to make decision for invasive planning or medical treatment, quickly and correctly. As mortality of patients that suffered from STEMI was considerable, we had to get these patients off our research.

## CONCLUSION

We can decide quickly in emergency room to distinguish patient derived a benefit from invasive strategies using TIMI score. Also, TIMI risk score can be an excellent predictor to determine the extension of CAD in patients with STEMI. As a result, we should determine TIMI for any patient enters the emergency room and this score should be recorded in discharge letters.
